# Work absence in parents of youth who self-harm

**DOI:** 10.1136/bmjment-2025-301833

**Published:** 2025-09-26

**Authors:** Moa Karemyr, Ester Gubi, Anna Ohlis, Gergö Hadlaczky, David Mataix-Cols, Clara Hellner, Ralf Kuja-Halkola, Johan Bjureberg

**Affiliations:** 1 Centre for Psychiatry Research, Department of Clinical Neuroscience, Karolinska Institutet & Stockholm Health Care Services, Region Stockholm, Stockholm, Sweden; 2 Department of Global Public Health, Karolinska Institutet, Stockholm, Sweden; 3 National Swedish Center for Suicide Research and Prevention, Department of Learning Management and Ethics, Karolinska Institutet, Stockholm, Sweden; 4 Department of Clinical Sciences, Faculty of Medicine, Lund University, Lund, Sweden; 5 Department of Medical Epidemiology and Biostatistics, Karolinska Institutet, Stockholm, Sweden

**Keywords:** Child & adolescent psychiatry, Suicide & self-harm

## Abstract

**Background:**

Self-harm is a prevalent health concern among youths, with significant psychosocial impacts on both youths and their parents. The aim of this study is to describe the impact of offspring self-harm on parental work absence.

**Methods:**

This cohort study included 176 472 mothers and 161 833 fathers of 17 726 youths with a first self-harm diagnosis between the years of 2006 and 2016 and 177 260 matched youths without self-harm. It examined work absence due to family leave to care for a sick child and sick leave, before and after the child’s self-harm. Data were sourced from Swedish population-based registers. Conditional Poisson regression, adjusting for potential confounders, was used to analyse associations between self-harm and work absence in parents of youth with and without self-harm after the self-harm event, as well as in parents of self-harming youth before and after the self-harm event.

**Results:**

Parents of both sexes experienced work absence. Nevertheless, mothers were particularly affected. Youth self-harm was linked to increased family leave (rate ratios: mothers 3.47 (95% CI 3.25 to 3.72), fathers 2.71 (2.47 to 2.98)) and sick leave (mothers 1.25 (1.20 to 1.31), fathers 1.25 (1.17 to 1.33)). Parents of affected youth took more family leave during the self-harm year compared with the previous year (mothers 1.65 (1.55 to 1.75), fathers 1.41 (1.29 to 1.54)), with no corresponding rise in sick leave.

**Conclusions:**

Parents of self-harming youths experience increased work absence, especially family leave, peaking around self-harm events. These results highlight the broader impact of youth self-harm on families and the need for support systems addressing both youth and caregiver well-being and work–life balance.

**WHAT IS ALREADY KNOWN ON THIS TOPIC**

- Youth self-harm has significant psychosocial effects on families, but its impact on parental work absence specifically had not been objectively quantified.

**WHAT THIS STUDY ADDS**

- This study, using Swedish population-based data, shows that parents of self-harming youth take more family and sick leave than other parents, with a sharp increase in family leave around the time of the self-harm event.

**HOW THIS STUDY MIGHT AFFECT RESEARCH, PRACTICE OR POLICY**

- These findings highlight the need for greater awareness in clinical practice of the broader family impact, and improved service development to provide targeted support for both youth and caregivers.

## Background

 Self-harm, defined as intentional self-poisoning or injury, irrespective of apparent purpose,^1^ is a common global health problem with a youth lifetime prevalence of 17%.^2^ Self-harm during youth is associated with a range of adverse outcomes, such as higher consumption of clinical care, more comorbid psychiatric diagnoses and lower global functioning compared with youth with other mental health issues.^3 4^ Further, self-harm constitutes one of the most robust risk factors for future suicide attempts.^5^

Research on youth self-harm has frequently focused on caregiver characteristics and parental behaviour.^6^,^8^ Specifically, parental psychopathology,^8^ a personal history of self-harm or suicide attempt,^7 9^ and socioeconomic factors such as low parental income during childhood^10^ have been highlighted as important risk factors of youth self-harm. However, youth self-harm also affects parents.^11^ Qualitative reports describe emotional burden, social isolation and practical as well as financial problems following the onset of offspring self-harm,^12^ suggesting a potentially bidirectional association. Parents of self-harming youths report higher parental stress compared with both clinical and non-clinical controls, with mothers experiencing more symptoms of anxiety, depression and stress compared with non-clinical controls.^13^ Additionally, mothers seem to seek psychiatric care more frequently in the immediate period following a self-harm event compared with the three preceding months.^14^ Finally, while representing the most severe outcome of self-harm, offspring suicide is associated with increased risk of long-term sick leave in parents.^15^

Parental involvement is an important component of psychosocial treatments for youth self-harm, with parents bearing the ultimate responsibility for ensuring their child’s safety.^16^,^18^ This responsibility can be highly demanding and sometimes requires parents to stay home, a situation described as incompatible with full-time work and leading to paid or unpaid work absence.^12^ In Sweden, social welfare provides partial compensation for taking leave of absence from work when due to either caring for a sick child or being ill oneself.^19 20^ Regardless, both caring for a sick child and personal sick leave are associated with a reduction in income. Prior research in this area has been mainly qualitative and cross-sectional.^11^ To the best of our knowledge, the impact of offspring self-harm on parental work absence has not been objectively quantified.

### Objective

The primary aim of the present population-based longitudinal cohort study was to investigate and compare work absence—operationalised as family leave to care for a sick child and sick leave—in parents of youth with or without self-harm after the first registered self-harm event. Secondarily, we aimed to compare work absence in parents of self-harming youth before and after the self-harm event.

## Methods

### Study design

We used a matched cohort design with data obtained from the Psychiatry Sweden register-linkage,^21^ which includes Swedish national administrative and clinical registers. In Sweden, all residents are assigned a personal identification number at birth or immigration, which allows for linkage across different registers. We included the following registries: The Swedish Social Insurance Agency Register, containing data on health insurance and parental insurance; The National Patient Register, with near 100% data coverage of psychiatric inpatient care from 1973 and 80% coverage for specialised outpatient care from 2001; The Longitudinal Integrated Database for Health Insurance and Labour Market Studies (LISA), containing data on health insurance, parental insurance, unemployment insurance, income and level of education since 1990; and The Total Population Register which includes demographic data such as region of residence, sex, civil status and family constellation since 1968.

### Cohort

The cohort consisted of 1 965 751 youths between the ages of 11 and 17 (born between 1988 and 2005) who were alive and residing in Sweden between 2006 and 2016, and their parents. Youths with no registered region of residence (n=1863) or no registered parent (n=8916) were excluded, as were adopted youths (n=19 520). We then identified youths who were diagnosed with self-harm (ICD codes X60–X84 and Y10–Y34; see online supplemental table S1) at any point during the study period (n=18 065). To allow for a minimum follow-up time of 1 day before and after the event, the self-harm had to have occurred between 2 January 2006 and 30 December 2016. Youths with several self-harm episodes were only included once, based on their first episode. Those with missing information about parental income or education during the year of inclusion or the year before inclusion (n=3461) were excluded, as were youth with a self-harm diagnosis who lacked information on region of residence at the time of receiving the diagnosis (n=35). Finally, three youths with a self-harm diagnosis were excluded because it was not possible to match them to 10 youths without a self-harm diagnosis.

Thus, the population that the matching was based on consisted of 1 931 991 youths (n=17 726 with a self-harm diagnosis). Using frequency matching, 10 youths without self-harm were identified for each youth with a self-harm diagnosis. These were matched based on sex, birth year, region of residence in Sweden during the year of the self-harm event and foreign-born status. The final youth population consisted of 194 986 individuals, including 17 726 youths with a self-harm diagnosis and 177 260 without self-harm diagnosis.

Then, mothers and fathers of each youth were identified, with the goal to create clusters consisting of one parent of a youth with self-harm, and approximately 10 parents of youths without self-harm. Parents who had more than one youth included in the study could be included once per included offspring. For 4655 youths, only mothers could be identified. For 745 youths, only fathers could be identified. Thus, not all parents of self-harming youths had 10 matched parents. Parents in clusters where no parent of a self-harming youth could be identified were excluded (8870 mothers and 16 060 fathers), as were parents who were not available for follow-up (7221 mothers and 15 700 fathers) and parents who had missing data in either income or highest level of education (both used as proxies for socioeconomic status (SES); 2423 mothers and 1393 fathers). In total, 18 514 mothers and 33 153 fathers were excluded, leaving on average 9.6 mothers of youths without self-harm per mother of youth with self-harm and 9.3 fathers of youths without self-harm per father of youth with self-harm. The follow-up period ranged from a minimum of 1 day to a maximum of 2 years (24 months) before and after the date of the youth’s self-harm diagnosis or matching, following previous research.^14^ Parents were censored at the date of emigration, death, or end of follow-up.

### Exposure

Individuals were defined as exposed if they were a biological mother or father of a youth who was diagnosed with self-harm during the study period (2006–2016).

### Outcomes

We examined family leave to care for a sick child and sick leave in parents. Family leave data are available as number of days of financial claims for family leave per calendar year. We counted days with family leave during the calendar year the self-harm event occurred, as well as the year before and after, except for the individuals who had their event in 2006 and 2016. Sick leave data are available as number of days of financial claims for own sickness absence with dates for each day on sick leave. For sick leave, we counted days with sick leave during the 2 years preceding the self-harm event as well as the 2 years following. As for family leave, the follow-up time was limited by the range of the study period. For the primary aim, we compared days with family leave and sick leave, respectively, in parents of youth with self-harm and parents of youth without. For the secondary aim, we studied only parents of youth with self-harm before and after the self-harm event. Differences in family leave over time were estimated through comparing days on family leave during the year before the self-harm event with the year the self-harm occurred, as well as with the year after. Differences in sick leave over time were estimated through comparing days on sick leave 2 years before the self-harm event with the 2 years after.

### Covariates

Covariates included numbers of previous days of family leave (categorised as 0, 1–10, 11–30, 31–366) and sick leave (categorised as 0, 1–10, 11–30, 31–366, 367–730), parental SES (using income and highest level of education as proxies), any registered previous psychiatric disorder, parental age, as well as number of siblings aged <12 years (only included in analyses of family leave). For family leave, previous occurrence was defined as occurrence during the year before the self-harm event/matching date. For sick leave, previous occurrence was defined as occurrence during the 2 years before the self-harm event/matching date. For analyses of family leave, number of siblings aged <12 years was also included as a covariate since, until age 12, compensation by state-handled obligatory parental insurance for family leave to care of a sick child requires no medical certificate for shorter absence (up to 5 days in a row) or special handling/decision by the insurance agency/fund.

### Statistical analysis

Descriptive statistics were calculated for demographic characteristics. We summarised days of family leave and sick leave by year for mothers and fathers, respectively. Analyses were conducted comparing parents of youth with self-harm with parents of youth without self-harm, matched on youth characteristics. Mothers and fathers were analysed separately. For the primary aim, conditional Poisson regression models, conditioned on the matching cluster, were used to test between-group differences in rates of family and sick leave. For family leave, models included rates during the calendar year of the self-harm event as well as the year after. For sick leave, rates were compared during the follow-up time of up to 2 years following the self-harm event. To model rates, an offset variable based on the number of follow-up days available was included in analyses. Adjustments for potential confounders were done in two stages. In the first stage, the analyses were adjusted for the history of the respective outcome (categorised number of days of family and sick leave). In the second stage, we adjusted for history of the outcome, SES (categorised in quintiles for income and as primary school, secondary school and university for highest level of education), any registered parental psychiatric disorder before the self-harm event and parental age. For family leave, these adjustments also included the number of siblings aged <12 years. For the secondary aim, conditional Poisson regression models were used to test within-individual differences in rates of family leave and sick leave in parents of self-harming youth before and after the event. For family leave, rates during the calendar year before the self-harm event were compared with rates during the year of the self-harm event as well as the year after. For sick leave, rates during the 2 years before the self-harm event were compared with rates during the two following years. Cluster robust standard errors were obtained using robust sandwich estimator. Results were presented as rate ratios (RRs) with 95% CIs. Data management was performed using SAS (V.9.4) and R (V.4.3.1).^22^ Statistical analyses were conducted using the gnm package in R.^23^

## Findings

### Study cohort

For demographic characteristics of the study cohort, see table 1 for youth characteristics, table 2 for mother characteristics and table 3 for father characteristics. We included 16 706 mothers of youth with self-harm and 159 766 mothers of youth without self-harm, resulting in a total of 176 472 mothers, as well as 15 771 fathers of youth with self-harm and 146 062 fathers of youth without self-harm, resulting in a total of 161 833 fathers. From the date of the youth self-harm event or matching, parents were followed for up to 2 years (mean (SD); follow-up time was 688.7 (136.6) days for mothers and 687.2 (139.3) days for fathers). The mean (SD) youth age at the time of the first registered self-harm was 15.6 (1.6) years for females and 15.3 (1.9) years for males. For parents, the mean (SD) age at the time of youth self-harm or matching was 43.8 (5.5) years for mothers of youth with self-harm, 44.6 (5.3) years for mothers of youth without self-harm, 46.9 (6.6) years for fathers of youth with self-harm and 47.5 (6.3) years for fathers of youth without self-harm. The proportion of mothers of self-harming youth with >10 days of yearly family leave was consistently higher than for mothers of youth without self-harm, with a peak during the year of the self-harm event (11.7% vs 3.5%). While consistently lower proportions as compared with the mothers, the same pattern was observed for fathers (2.5% vs 1.1% during the year of self-harm). For sick leave, the proportion of mothers of self-harming youth with >10 days of sick leave was consistently higher than for mothers of youth without self-harm, with no peak following the self-harm event (27.9% vs 21.1% during the 2 years before the self-harm, 26.6% vs 19.3% during the 2 years after). While the proportion on sick leave was lower among fathers, the differences between fathers of youth with and without self-harm matched the pattern for mothers (16.8% vs 13.0% during the 2 years before the self-harm, 15.7% vs 12% during the 2 years after). Finally, registered psychiatric diagnoses were twice as common among the parents of self-harming youth.

**Table 1:** Study and youth characteristics

Characteristic	No. (%) With self-harm	No. (%) Without self-harm	No. (%) Total
Youth, no. (%)	17 726 (100)	177 260 (100)	194 986 (100)
Sex			
Female	11 379 (64.2)	113 790 (64.2)	125 169 (64.2)
Male	6347 (35.8)	63 470 (35.8)	69 817 (35.8)
Age at exposure/matching date, mean (SD) years			
Total	15.4 (1.7)	15.5 (1.8)	15.50 (1.8)
Female	15.6 (1.6)	15.6 (1.7)	15.60 (1.7)
Male	15.3 (1.9)	15.3 (2.0)	15.30 (1.9)
Foreign-born status			
Born in Sweden	16 583 (93.6)	165 830 (93.6)	182 413 (93.6)
Foreign-born	11 430 (6.5)	1143 (6.5)	12 573 (6.5)
Psychiatric comorbidity*†			
Anxiety, OCD and stress-related disorders	2772 (15.6)	3364 (1.9)	6136 (3.1)
Depressive disorders	2542 (14.3)	2007 (1.1)	4549 (2.3)
Non-affective and affective psychotic disorders	259 (1.5)	192 (0.1)	452 (0.0)
Personality disorders	185 (1.0)	132 (0.1)	317 (0.0)
ADHD and autism	1768 (10.0)	4785 (2.7)	6553 (0.3)
Eating disorders	602 (3.4)	1046 (0.6)	1648 (0.1)
Alcohol and substance use disorders	545 (3.1)	447 (0.3)	992 (0.1)

* Registered diagnostic codes according to the International Statistical Classification of Diseases and Related Health Problems-10th Revision (ICD-10), including anxiety, OCD and stress-related disorders (F40–45, F48); depressive disorders (F32–34, F38–39, except F32.3, F33.3); non-affective and affective psychotic disorders (ICD-10: F20–24, F28–29; F25, F30–31, F32.3, F33.3); personality disorders (F60–63, F68–69); ADHD and autism (F90, F84); eating disorders (F50); alcohol and substance use disorders (F10.1–10.9; F11–19, except F17).

† Registered prior to self-harm/matching date.

ADHD, attention deficit hyperactivity disorder; OCD, obsessive-compulsive disorder.

**Table 2:** Mother characteristics

Characteristic	No. (%) With self-harm	No. (%) Without self-harm	No. (%) Total
Mothers, no. (%)	16 706 (9.5)	159 766 (90.5)	176 472 (100)
Mean (SD) follow-up days	688.28 (137.4)	688.75 (136.6)	688.70 (136.6)
Family leave year before self-harm, no. days			
0	12 021 (72.0)	119 156 (74.6)	131 177 (74.3)
1–10	3409 (20.4)	33 579 (21.0)	36 988 (21.0)
11–30	950 (5.7)	6153 (3.9)	7103 (4.0)
31–365	326 (2.0)	878 (0.6)	1204 (0.7)
Family leave year of self-harm, no. days			
0	11 618 (69.5)	129 077 (80.8)	140 695 (79.7)
1–10	3134 (18.8)	25 063 (15.7)	28 197 (16.0)
11–30	1241 (7.4)	4781 (3.0)	6022 (3.4)
31–365	713 (4.3)	845 (0.5)	1558 (0.9)
Family leave year after self-harm, no. days			
0	13 422 (80.3)	136 180 (85.2)	149 602 (84.8)
1–10	2252 (13.5)	19 229 (12.0)	21 481 (12.2)
11–30	686 (4.1)	3638 (2.3)	4324 (2.5)
31–365	346 (2.1)	719 (0.5)	1065 (0.6)
Sick leave 2 years before self-harm, no. days			
0	11 801 (70.6)	131 230 (77.8)	143 031 (77.2)
1–10	245 (1.5)	1860 (1.1)	2105 (1.1)
10–30	988 (5.9)	8743 (5.2)	9731 (5.3)
30–365	2925 (17.5)	21 651 (12.8)	24 576 (13.3)
366–730	747 (4.5)	5152 (3.1)	5899 (2.2)
Sick leave 2 years after self-harm, no. days			
0	12 079 (72.3)	134 551 (79.8)	146 630 (79.1)
1–10	182 (1.1)	1538 (0.9)	1720 (0.9)
10–30	907 (5.4)	8022 (4.8)	8929 (4.8)
30–365	2838 (17.0)	20 451 (12.1)	23 289 (12.6)
366–730	700 (4.2)	4074 (2.4)	4774 (2.6)
Age at exposure/matching date, mean, SD years	43.8 (5.5)	44.6 (5.3)	44.5 (5.4)
Highest level of education			
Primary school	2550 (15.3)	17 850 (11.2)	20 400 (11.6)
Secondary school	8786 (52.6)	81 117 (50.8)	89 903 (50.9)
University	5370 (32.1)	60 799 (38.1)	66 169 (37.5)
Yearly disposable income, divided in quintiles			
Quintile 0, low	6479 (38.8)	51 056 (32)	57 535 (32.6)
Quintile 1	4768 (28.5)	45 199 (28.3)	49 967 (28.3)
Quintile 2, medium	3101 (18.6)	33 646 (21.1)	36 747 (20.8)
Quintile 3	1519 (9.1)	18 759 (11.7)	20 278 (11.5)
Quintile 4, high	839 (5.0)	11 106 (7.0)	11 945 (6.8)
Any prior psychiatric diagnosis*	3510 (21.0)	15 901 (10.0)	19 411 (11.0)

* Registered diagnostic codes according to the International Statistical Classification of Diseases and Related Health Problems-10th Revision (ICD-10), including anxiety, OCD and stress-related disorders (F40–45, F48); depressive disorders (F32–34, F38–39, except F32.3, F33.3); non-affective and affective psychotic disorders (F20–24, F28–29; F25, F30–31, F32.3, F33.3); personality disorders (F60–63, F68–69); ADHD and autism (F90, F84); eating disorders (F50); alcohol and substance use disorders (F10.1–10.9; F11–19, except F17).

ADHD, attention deficit hyperactivity disorder; OCD, obsessive-compulsive disorder.

**Table 3:** Father characteristics

Characteristic	No. (%) With self-harm	No. (%) Without self-harm	No. (%) Total
Fathers, no. (%)	15 771 (9.8)	146 062 (90.3)	161 833 (100)
Mean (SD) follow-up days	685.58 (141.8)	687.38 (139.0)	687.2 (139.3)
Family leave year before self-harm, no. days			
0	12 939 (82.0)	122 951 (84.2)	135 890 (84.0)
1–10	2343 (14.9)	20 395 (14.0)	22 738 (14.1)
11–30	398 (2.5)	2403 (1.7)	2801 (1.7)
31–365	91 (0.6)	313 (0.2)	404 (0.3)
Family leave year of self-harm, no. days			
0	12 990 (82.4)	128 934 (88.3)	141 924 (87.7)
1–10	2058 (13.1)	14 952 (10.2)	17 010 (10.5)
11–30	516 (3.3)	1912 (1.3)	2428 (1.5)
31–365	207 (1.3)	264 (0.2)	471 (0.3)
Family leave year after self-harm, no. days			
0	13 943 (88.4)	133 367 (91.3)	147 310 (91.3)
1–10	1440 (9.13)	11 081 (7.6)	12 521 (7.3)
11–30	284 (1.8)	1386 (0.9)	1670 (1.0)
31–365	104 (0.7)	228 (0.2)	332 (0.2)
Sick leave 2 years before self-harm, no. days			
0	13 003 (82.5)	139 769 (86.2)	152 772 (85.9)
1–10	126 (0.8)	1296 (0.8)	1422 (0.8)
10–30	673 (4.3)	5816 (3.6)	6489 (3.6)
30–365	1633 (10.4)	12 921 (8.0)	14 554 (8.2)
366–730	336 (2.1)	2320 (1.4)	2656 (1.5)
Sick leave 2 years after self-harm, no. days			
0	13 192 (83.6)	141 680 (87.4)	154 872 (87.1)
1–10	124 (0.8)	1059 (0.7)	1183 (0.7)
10–30	564 (3.6)	5285 (3.3)	5849 (3.3)
30–365	1600 (10.2)	12 214 (7.5)	13 814 (7.8)
366–730	291 (1.9)	1884 (1.2)	2175 (1.2)
Age at exposure/matching date, mean, SD years	46.9 (6.6)	47.5 (6.3)	47.5 (6.3)
Highest level of education			
Primary school	3037 (19.3)	22 651 (15.5)	25 688 (15.9)
Secondary school	8882 (56.3)	78 326 (53.6)	87 208 (53.9)
University	3852 (24.4)	45 085 (30.9)	48 937 (30.2)
Yearly disposable income, divided in quintiles			
Quintile 0, low	4387 (27.8)	35 830 (24.5)	40 217 (24.9)
Quintile 1	3823 (24.2)	36 689 (25.1)	40 512 (25.0)
Quintile 2, medium	3178 (20.2)	32 003 (21.9)	35 181 (21.7)
Quintile 3	2227 (14.1)	21 672 (14.8)	23 899 (14.8)
Quintile 4, high	2156 (13.7)	19 868 (13.6)	22 024 (13.6)
Any prior psychiatric diagnosis*	2757 (17.5)	11 530 (7.9)	14 287 (8.8)

* Registered diagnostic codes according to the International Statistical Classification of Diseases and Related Health Problems 10th Revision (ICD-10), including anxiety, OCD and stress-related disorders (F40–45, F48); depressive disorders (F32–34, F38–39, except F32.3, F33.3); non-affective and affective psychotic disorders (F20–24, F28–29; F25, F30–31, F32.3, F33.3); personality disorders (F60–63, F68–69); ADHD and autism (F90, F84); eating disorders (F50); alcohol and substance use disorders (F10.1–10.9; F11–19, except F17).

ADHD, attention deficit hyperactivity disorder; OCD, obsessive-compulsive disorder.

### Work absence in parents of youth with and without self-harm

Results are presented in table 4. Parents of youth with self-harm had higher rates of family leave the year during which the self-harm/matching occurred. Fully adjusted RRs were 3.47 (95% CI 3.25, 3.72) for mothers and 2.71 (95% CI 2.47, 2.98) for fathers. The year after the self-harm occurred, fully adjusted RRs were 2.49 (95% CI 2.28, 2.73) for mothers and 2.05 (95% CI 1.83, 2.29) for fathers. Higher rates in parents of youth with self-harm were also observed for sick leave, with fully adjusted RRs of 1.25 (95% CI 1.20, 1.31) for mothers and 1.25 (95% CI 1.17, 1.33) for fathers.

**Table 4:** Results from conditional Poisson regression comparing parents of youth with and without self-harm, as well as parents of youth with self-harm before and after the event

Outcome	RR (95% CI) Crude	P	RR (95% CI) Adjusted*	P	RR (95% CI) Adjusted†	P
Family leave						
Comparisons between parents of youth with and without self-harm						
Mothers						
Year of self-harm	3.71 (3.51, 3.93)	<0.001	3.42 (3.21, 3.65)	<0.001	3.47 (3.25, 3.72)	<0.001
Year after self-harm	2.62 (2.42, 2.83)	<0.001	2.45 (2.24, 2.68)	<0.001	2.49 (2.28, 2.73)	<0.001
Fathers						
Year of self-harm	2.72 (2.51, 2.95)	<0.001	2.69 (2.45, 2.94)	<0.001	2.71 (2.47, 2.98)	<0.001
Year after self-harm	2.03 (1.84, 2.24)	<0.001	2.03 (1.82, 2.27)	<0.001	2.05 (1.83, 2.29)	<0.001
Comparisons in parents of youth with self-harm before and after the event‡						
Mothers						
Year of self-harm	1.65 (1.55, 1.75)	<0.001	NA	NA	NA	NA
Year after self-harm	1.03 (0.94, 1.13)	0.492	NA	NA	NA	NA
Fathers						
Year of self-harm	1.41 (1.29, 1.54)	<0.001	NA	NA	NA	NA
Year after self-harm	0.89 (0.80, 0.99)	0.035	NA	NA	NA	NA
Sick leave						
Comparisons between parents of youth with and without self-harm						
Mothers	1.57 (1.51, 1.64)	<0.001	1.34 (1.29, 1.40)	<0.001	1.25 (1.20, 1.31)	<0.001
Fathers	1.49 (1.40, 1.59)	<0.001	1.36 (1.27, 1.45)	<0.001	1.25 (1.17, 1.33)	<0.001
Comparisons in parents of youth with self-harm before and after the event§						
Mothers	1.02 (0.97, 1.07)	0.435	NA	NA	NA	NA
Fathers	0.98 (0.92, 1.05)	0.630	NA	NA	NA	NA

* Adjusted for previous family leave and sick leave, respectively.

† Adjusted for previous family leave or sick leave, socioeconomic status, parental age, presence of any psychiatric diagnosis before self-harm/matching date, as well as siblings aged <12 years (family leave only).

‡ The reference level is the calendar year before the registered self-harm event.

§ The reference level is the 2 years before the registered self-harm event.

RR, rate ratio.

### Work absence in parents of youth with self-harm before and after the self-harm event

Results are presented in table 4. Within-individual analyses demonstrated increased rates of family leave in the calendar year of the self-harm event, when compared with the year prior to the event in both mothers (RR=1.65, 95% CI 1.55, 1.75) and fathers (RR=1.41, 95% CI 1.29, 1.54). The year after the event, the rates decreased to levels comparable to the rates prior to the event in mothers (RR=1.03, 95% CI 0.94, 1.13) and to levels lower than the rates observed prior to the event in fathers (RR=0.89, 95% CI 0.80, 0.99). However, no increased rates were identified in sick leave during the 2 years following the self-harm event when compared with the 2 years prior to the event, neither among mothers (RR=1.02, 95% CI 0.97, 1.07) nor fathers (RR=0.98, 95% CI 0.92, 1.05).

## Discussion

To the best of our knowledge, this is the first study that provides a comprehensive longitudinal assessment of objectively recorded work absence in parents of youth engaging in self-harm. Combining between-group and within-individual designs, this cohort study investigated the associations between parent and youth while also adjusting for confounding caused by stable factors shared in the families. The findings demonstrate that youth self-harm is associated with increased risk of both family leave and sick leave among parents. Moreover, parents of youth with self-harm experienced a temporary acute rise in family leave during the year of the self-harm incident. This temporal association was, however, not observed for sick leave. These results align with earlier qualitative and cross-sectional findings, confirming the parental burden associated with youth self-harm.^11^ Furthermore, we expand on previous longitudinal findings of increased psychiatric care-seeking patterns following offspring self-harm^14^ by studying work absence as another indicator of psychosocial burden. The national population-based registers available in Sweden offer a unique opportunity to use objectively recorded health insurance data to estimate parents’ time off work. While the increased work absence observed among parents of self-harming youths was anticipated, given clinical recommendations^16^,^18^ and qualitative reports,^12^ its magnitude has not been previously quantified. The identified temporal association of youth self-harm and family leave highlights the potential practical and financial impact of parents acting as protective safety net for their child. In Sweden, publicly funded social insurance provides partial financial compensation for family and sick leave. This support is not universally available, and in some parts of the world, such work absences may lead to complete withdrawal from the job market. Given that low parental income during childhood is a known risk factor for adolescent self-harm,^24^ this finding holds considerable importance. While youth self-harm was associated with increased work absence for parents of both sexes, mothers were more affected. Women in general have a higher risk of sick leave compared with men, which might be explained in regards to differences in different domains such as prevalence of psychiatric disorders, healthcare-seeking patterns and caring for children.^25^ Indeed, previous studies have similarly found larger effects of youth self-harm on mothers, in regards to both reports of stress, depression and anxiety^13^ as well as psychiatric care seeking.^14^

In line with previous research,^3^ youths who engaged in self-harm had more comorbid psychiatric disorders than those without self-harm. As expected, given in previous research,^8 13 14^ a similar pattern was observed among their parents: the proportions of both mothers and fathers with any registered psychiatric disorder were twice as high as those among parents of youths with no evidence of self-harm. This finding may help explain the observed differences in sick leave, as psychiatric disorders are a leading cause of sick leave.^26^ Furthermore, familial risk factors shared between psychosocial functioning and self-harming behaviours^27^,^29^ might confound the association between youth self-harming behaviour and parental work absence, thus motivating the need for the within-individual design of the secondary aim. Although risk factors shared between parent and offspring might partially explain the increased risk of work absence in parents of self-harming youth, within-individual comparisons suggest that work absence due to family leave indeed is temporally associated with the self-harm event.

### Limitations

This cohort study has some limitations. First, the register-based design is limited to using healthcare-registered self-harm diagnoses to determine exposure status. As a result, only youths who engaged with the healthcare system and whose self-harm was diagnosed were included. It is likely that the included sample represents a subpopulation of self-harming youths engaging in more severe forms of self-harm, such as intoxication or cutting, requiring healthcare. Furthermore, indications of the reason for family leave to care for a sick child or even for which child the compensation was for (in families with more than one child) were not available in the registers used in the current study. Consequently, it is possible that some registered absences were unrelated to self-harm. However, the number of siblings was included as a covariate to help account for some of this uncertainty. Moreover, data on family leave lack specific dates for each period; hence our reporting of the total number of days on a year-to-year basis. The resolution for this outcome is therefore less fine-grained than for sick leave. Additionally, the register only contained data on sick leave periods lasting over 14 days. Thus, shorter periods of work absence due to sick leave are not captured in this study and the sick leave category of 1–10 days over the year reported in tables2  3 includes individuals with either part time sick leave over a longer period (registered as whole days) or individuals whose sick leave period either started before the 1 January or ended after the 31 December. Finally, specific effects on minority groups such as families with adopted youths or same-sex parents have not been identified in this study. Extension of this work might include and report data on families with more diverse backgrounds and family constellations.

### Clinical implications

This cohort study showed that parents of self-harming youth were more likely to be absent from work than other parents, and that youth self-harm events were associated with temporary increases in parental work absenteeism related to caring for a child. The findings highlight the broader impact of youth self-harm on family functioning, emphasising the need for support systems that address not only the affected youth but also their caregivers' well-being and work–life balance.

## Supplementary material

10.1136/bmjment-2025-301833 online supplemental file 1

## Data Availability

Data are available upon reasonable request.
