# Supplementary material for: Work absence in parents of youth who self-harm
Source: BMJ Ment Health. 2025 Sep 26;28(1):e301833. doi: 10.1136/bmjment-2025-301833 (PMC12481354; doi:10.1136/bmjment-2025-301833)
Supplement: online supplemental file 1 [file bmjment-28-1-s001.docx]

**Supplement**

[**Table S1.**  **Specifications of diagnostic codes X60-84 and Y10-34 from the International Statistical Classification of Diseases and Related Health Problems - 10th Revision (ICD-10)** 2](#_Toc193801321)

| **Table S1.**  **Specifications of diagnostic codes X60-84 and Y10-34 from the International Statistical Classification of Diseases and Related Health Problems - 10th Revision (ICD-10)** | |
| --- | --- |
| ICD-10 codes | Specification |
| X60 | Intentional self-poisoning by and exposure to nonopioid analgesics, antipyretics and antirheumatics |
| X61 | Intentional self-poisoning by and exposure to antiepileptic, sedative-hypnotic, antiparkinsonism and psychotropic drugs, not elsewhere classified |
| X62 | Intentional self-poisoning by and exposure to narcotics and psychodysleptics (hallucinogens), not elsewhere classified |
| X63 | Intentional self-poisoning by and exposure to other drugs acting on the autonomic nervous system |
| X64 | Intentional self-poisoning by and exposure to other and unspecified drugs, medicaments and biological substances |
| X65 | Intentional self-poisoning by and exposure to alcohol |
| X66 | Intentional self-poisoning by and exposure to organic solvents and halogenated hydrocarbons and their vapours |
| X67 | Intentional self-poisoning by and exposure to carbon monoxide and other gases and vapours |
| X68 | Intentional self-poisoning by and exposure to pesticides |
| X69 | Intentional self-poisoning by and exposure to other and unspecified chemicals and noxious substances |
| X70 | Intentional self-harm by hanging, strangulation and suffocation |
| X71 | Intentional self-harm by drowning and submersion |
| X72 | Intentional self-harm by handgun discharge |
| X73 | Intentional self-harm by rifle, shotgun and larger firearm discharge |
| X74 | Intentional self-harm by other and unspecified firearm discharge |
| X75 | Intentional self-harm by explosive material |
| X76 | Intentional self-harm by smoke, fire and flames |
| X77 | Intentional self-harm by steam, hot vapours and hot objects |
| X78 | Intentional self-harm by sharp object |
| X79 | Intentional self-harm by blunt object |
| X80 | Intentional self-harm by jumping from a high place |
| X81 | Intentional self-harm by jumping or lying before moving object |
| X82 | Intentional self-harm by crashing of motor vehicle |
| X83 | Intentional self-harm by other specified means |
| X84 | Intentional self-harm by unspecified means |
| Y10 | Poisoning by and exposure to nonopioid analgesics, antipyretics and antirheumatics, undetermined intent |
| Y11 | Poisoning by and exposure to antiepileptic, sedative-hypnotic, antiparkinsonism and psychotropic drugs, not elsewhere classified, undetermined intent |
| Y12 | Poisoning by and exposure to narcotics and psychodysleptics [hallucinogens], not elsewhere classified, undetermined intent |
| Y13 | Poisoning by and exposure to other drugs acting on the autonomic nervous system, undetermined intent |
| Y14 | Poisoning by and exposure to other and unspecified drugs, medicaments and biological substances, undetermined intent |
| Y15 | Poisoning by and exposure to alcohol, undetermined intent |
| Y16 | Poisoning by and exposure to organic solvents and halogenated hydrocarbons and their vapours, undetermined intent |
| Y17 | Poisoning by and exposure to carbon monoxide and other gases and vapours, undetermined intent |
| Y18 | Poisoning by and exposure to pesticides, undetermined intent |
| Y19 | Poisoning by and exposure to other and unspecified chemicals and noxious substances, undetermined intent |
| Y20 | Hanging, strangulation and suffocation, undetermined intent |
| Y21 | Drowning and submersion, undetermined intent |
| Y22 | Handgun discharge, undetermined intent |
| Y23 | Rifle, shotgun and larger firearm discharge, undetermined intent |
| Y24 | Other and unspecified firearm discharge, undetermined intent |
| Y25 | Contact with explosive material, undetermined intent |
| Y26 | Exposure to smoke, fire and flames, undetermined intent |
| Y27 | Contact with steam, hot vapours and hot objects, undetermined intent |
| Y28 | Contact with sharp object, undetermined intent |
| Y29 | Contact with blunt object, undetermined intent |
| Y30 | Falling, jumping or pushed from a high place, undetermined intent |
| Y31 | Falling, lying or running before or into moving object, undetermined intent |
| Y32 | Crashing of motor vehicle, undetermined intent |
| Y33 | Other specified events, undetermined intent |
| Y34 | Unspecified event, undetermined intent |
| Abbreviations: ICD-10, International Statistical Classification of Diseases and Related Health Problems - 10th Revision | |
